# Protection Against XY Gonadal Sex Reversal by a Variant Region on Mouse Chromosome 13

**DOI:** 10.1534/genetics.119.302786

**Published:** 2019-12-13

**Authors:** Catherine Livermore, Michelle Simon, Richard Reeves, Isabelle Stévant, Serge Nef, Madeleine Pope, Ann-Marie Mallon, Sara Wells, Nick Warr, Andy Greenfield

**Affiliations:** * Mammalian Genetics Unit, MRC Harwell Institute, Oxfordshire, OX11 0RD, UK; † Department of Genetic Medicine and Development, University of Geneva Medical School, CH 1211 Geneva 4, Switzerland; ‡ The Mary Lyon Centre, MRC Harwell Institute, Oxfordshire, OX11 0RD, UK

**Keywords:** Sex determination, testis, ovary, Y chromosome, ovotestis, Genetics of Sex

## Abstract

XY C57BL/6J (B6) mice harboring a *Mus musculus domesticus*-type Y chromosome (Y*^POS^*), known as B6.Y*^POS^* mice, commonly undergo gonadal sex reversal and develop as phenotypic females. In a minority of cases, B6.Y*^POS^* males are identified and a proportion of these are fertile. This phenotypic variability on a congenic B6 background has puzzled geneticists for decades. Recently, a B6.Y*^POS^* colony was shown to carry a non-B6-derived region of chromosome 11 that protected against B6.Y*^POS^* sex reversal. Here. we show that a B6.Y*^POS^* colony bred and archived at the MRC Harwell Institute lacks the chromosome 11 modifier but instead harbors an ∼37 Mb region containing non-B6-derived segments on chromosome 13. This region, which we call *Mod13*, protects against B6.Y*^POS^* sex reversal in a proportion of heterozygous animals through its positive and negative effects on gene expression during primary sex determination. We discuss *Mod13*’s influence on the testis determination process and its possible origin in light of sequence similarities to that region in other mouse genomes. Our data reveal that the B6.Y*^POS^* sex reversal phenomenon is genetically complex and the explanation of observed phenotypic variability is likely dependent on the breeding history of any local colony.

IN mammals, the Y chromosome is a dominant male determinant due to the presence of the *SRY* gene. SRY’s occupancy of key enhancers causes the upregulation of *SOX9*, which itself drives the differentiation of Sertoli cells and the formation of testis cords (Sekido and Lovell-Badge 2008; Gonen *et al.* 2018). In XX gonads, in the absence of SRY, other pathways, including canonical WNT signaling and the transcription factor FOXL2, induce ovary development (Pannetier *et al.* 2016). Many years of study in the mouse model system indicate that the timing of *Sry* expression is crucial in testis development: any significant reduction or delay in *Sry* expression during a critical “window of opportunity” for testis determination may result in gonadal sex reversal (*i.e.*, XY ovary or ovotestis development), due to a failure to inhibit the activity of pro-ovarian genes (Hiramatsu *et al.* 2008). Mutations that disrupt molecules required for normal spatiotemporal expression of *Sry* can cause XY gonadal sex reversal due to such a delay [reviewed in Carré and Greenfield (2014); Larney *et al.* (2014)]. However, the exact mechanisms by which this diverse class of molecules regulate *Sry* expression remain unclear.

One model system for examining the role of *Sry* in sex determination, and its interaction with genes on the autosomes, is B6.Y*^DOM^* sex reversal. The C57BL6/J (B6) genetic background is sensitized to disruptions to testis determination due to precocious pro-ovarian gene expression profiles during the sex-determining period of gonadogenesis (Munger *et al.* 2009; Correa *et al.* 2012). Introgression of the Y chromosome from certain *Mus musculus domesticus* strains into the B6 genetic background (B6.Y*^DOM^*) disrupts testis determination to varying degrees. One classic example involves the wild-derived *M. d. poschiavinus* Y chromosome: when on the B6 genetic background (B6.Y*^POS^*), where all autosomes and the X chromosome are predicted to be B6-derived, XY female development is the norm and examination of B6.Y*^POS^* fetal gonad development reveals XY ovary or ovotestis development as the commonest developmental outcomes (Eicher *et al.* 1982, 1996). While differences exist in the SRY protein isoforms encoded by distinct Y*^DOM^* alleles, these cannot alone account for the different phenotypic outcomes of Y*^DOM^* chromosome introgression (Albrecht and Eicher 1997; Albrecht *et al.* 2003). Rather, it has been reported that altered expression of the relevant *Sry^DOM^* allele also plays a role (Nagamine *et al.* 1999). The peak of *Sry^POS^* expression has been reported to be delayed in B6.Y*^POS^* gonads, when compared to peak expression of *Sry^B6^* (Bullejos and Koopman 2005).

With respect to B6.Y*^POS^*, a complex model of sex reversal has emerged from a number of studies, invoking misregulation of *Sry* expression as a consequence of a mismatch between B6 autosomal and/or X-linked gene products required for its normal spatiotemporal expression and regulatory elements of the *Sry* locus; and altered stability and/or functionality of the SRY^DOM^ protein isoforms in the context of the B6 genome. The exact molecular basis of the proposed functional mismatches remains unclear, as do the molecular events underlying *Sry*’s unique spatiotemporal profile of expression. Understanding has been hampered by the use, over many years, of different techniques to examine and quantify gene expression. Indeed, recent studies have called into question whether the B6.Y*^POS^* genome can be considered a unitary genetic background in the traditional sense.

The degree of gonadal sex reversal is typically variable between B6.Y*^POS^* individuals. Around 70–75% of XY individuals develop as phenotypic females and 25–30% as males. B6.Y*^POS^* males (once classified as hermaphrodites) have varying amounts of testicular tissue in either gonad, but enough to masculinize external genitalia. Given that any B6.Y*^POS^* colony is maintained by crossing fertile males to B6 females at each generation, the basis of this phenotypic variability has been unclear. Recently, a dominantly acting, autosomal modifier of B6.Y*^POS^* sex reversal, which protects against sex reversal, was reported in a United States colony of this strain (Arboleda *et al.* 2014). The 4.5 Mb region of chromosome 11 was identified by virtue of it not being B6-derived: instead, it is thought to derive from the *M. d. poschiavinus* (POS A) genome that was originally used to establish the B6.Y^POS^ strain. The region in question encompasses the regulatory sequences upstream of the *Sox9* gene and is thought to promote *Sox9* expression, even in the presence of the “weaker” *Sry^POS^* allele, thus protecting against B6.Y*^POS^* sex reversal. This, in turn, is thought to account for its persistence in the B6.Y*^POS^* colony in question: at each generation, fertile males selected for further breeding are highly likely to be heterozygous for this region because in its absence XY female, or infertile hermaphrodite development, will most likely ensue.

Here, we report an analysis of the genetics of sex reversal in a B6.Y*^POS^* colony rederived from the frozen embryo archive at Harwell, which was originally investigated in the 1980–90s before archiving. This colony exhibits familiar ratios of XY female and male development, but lacks the chromosome 11 modifier locus. Instead, whole-genome sequence analysis of a B6.Y*^POS^* stud male reveals heterozygosity across an ∼37 Mb region of chromosome 13 for segments not derived from B6. This region, termed *Mod13*, is of unknown origin and confers protection against gonadal sex reversal in the fetal period by positively affecting *Sry* expression and negatively affecting pro-ovarian genes; but it contains no known primary sex-determining genes in its non-B6-derived segments. Our data reveal that B6.Y*^POS^* colonies in different parts of the world, if maintained in the absence of an *Sry* transgene or similar, may contain distinct modifiers that protect against sex reversal in a significant proportion of XY animals, depending on breeding history. We discuss the significance of these data for our understanding of B6.Y*^POS^* sex reversal.

## Materials and Methods

### Mouse strains

All mouse experimentation was approved by the Animal Welfare and Ethical Review Body at MRC Harwell. Mice used were bred with licensed approval from the UK Home Office (PPL 70/8898). Mice were housed in individually ventilated cages in a specific pathogen-free environment. Further details of micro- and macroenvironmental conditions are available on request. All lines were maintained on C57BL/6J (B6). The use of the name *M. d. poschiavinus* is contested (Silver 1995); phylogenetic studies place this strain firmly within the *M. m. domesticus* fold. However, for the sake of consistency with the existing literature on the Y chromosome from this strain and its effect on testis determination, we continue to use *M. d. poschiavinus* (and B6.Y*^POS^*) here. Genotyping of *Mod13* single nucleotide polymorphisms (SNPs) was performed by analysis of SNPs listed in Supplemental Material, Table S2.

### Generation of embryos

Noon on the day of the copulatory plug was counted as 0.5 days *post coitum* (dpc). Adult mice were humanely killed by dislocation of the neck, confirmed by palpation, and embryos were decapitated in ice-cold, phosphate-buffered saline solution. Embryos collected at 11.5 dpc were staged accurately based on the number of tail somites (ts).

### Whole-mount *in situ* hybridization

Whole-mount *in situ* hybridization (WMISH) analysis of embryonic tissues and probes for *Sry*, *Sox9*, *Insl3*, and *Stra8* have been previously described (Bogani *et al.* 2009; Warr *et al.* 2009). At least three independent biological samples from a given group were analyzed with a particular marker.

### Quantitative RT-PCR

Total RNA was extracted using RNeasy plus micro kit (QIAGEN, Valencia, CA) from gonads separated from the mesonephros. Reverse transcription was carried out with 250 ng of total RNA using the High Capacity cDNA RT Kit (Applied Biosystems, Foster City, CA). Quantitative RT-PCR (qRT-PCR) was performed with Fast SYBR Green Master Mix (Life Technologies) on a 7500 Fast Real-Time PCR system (Applied Biosystems). RNA expression levels were normalized to those of *Hrpt1* (endogenous control) using the ΔΔCt method (Livak and Schmittgen 2001). At least three samples for each genotype were analyzed. Primer sequences are available on request.

### Whole-genome sequencing and bioinformatic analyses

For sequencing of a B6.Y^POS^ stud male, DNA was extracted from mouse tail tissue and sequencing (Illumina HiSeq2000) performed at the Wellcome Trust Centre for Human Genetics High Throughput Genomics Facility (Oxford, UK). This generated a mean read-depth of 13× from a single lane of 100 nucleotide paired-end reads. Reads were aligned to the reference genome (NCBIM38/mm10) using BWA (Li and Durbin 2009). Detection of SNPs in the alignment was made using a customized version of the Genome Analysis Toolkit (DePristo *et al.* 2011) with default parameters. We adopted a filtering strategy to identify high-confidence SNPs and reduce the incidence of false positives. SNP sites that failed the Genome Analysis Toolkit internal status check and had a mapping quality <200 were removed.

### Data availability

All reagents are available upon request. B6.Y^POS^ sequence data analyzed here have been submitted to the NCBI (BioSample accession: SAMN12896768). Supplemental material available at figshare: https://doi.org/10.25386/genetics.11323655.

## Results

### Identification of *Mod13* and its influence on B6.XY*^POS^* maleness

A B6.Y*^POS^* colony was bred at Harwell in the mid-1980s and subsequently archived in the 1990s. We have been unable to determine its precise origins, but B6.Y*^POS^* was originally generated in the United States (Eicher *et al.* 1982). Before this study, the colony had been maintained on B6 for ∼6 years, in the absence of any protective transgene, by selecting rare fertile stud males at each generation to cross with B6 females. Around 65–70% of XY mice were routinely scored as female at weaning.

**Figure 1:**
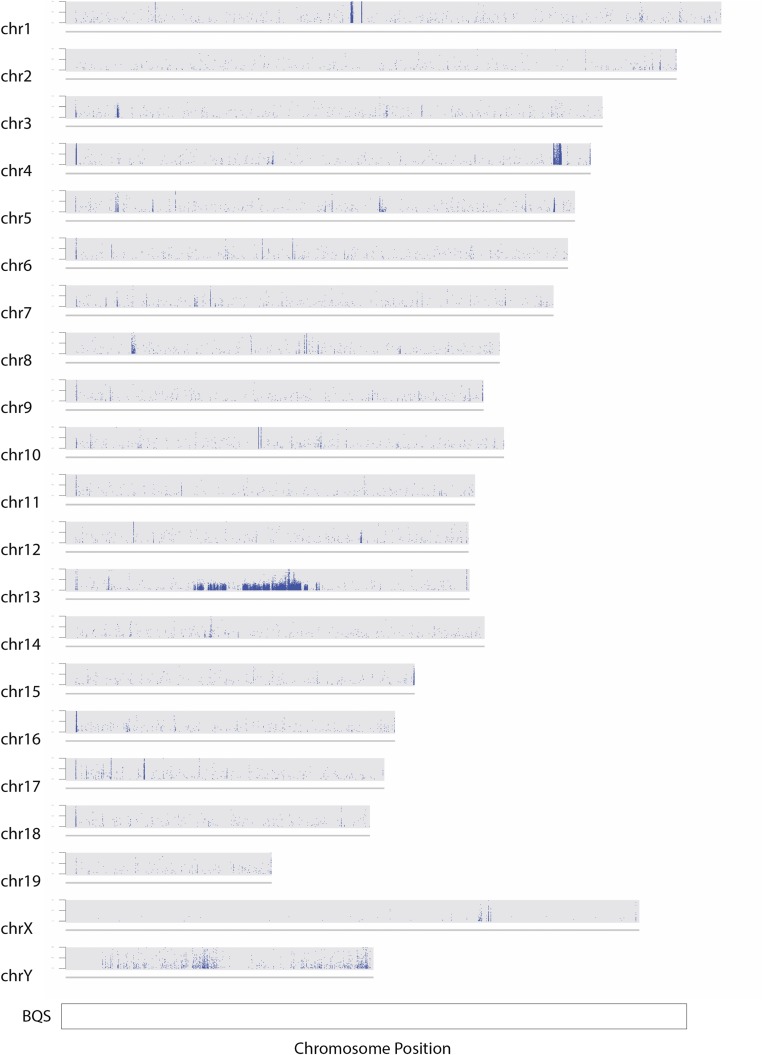
Identification of the *Mod13* variant region on chromosome 13. The distribution of SNPs in the B6.YPOS genome of a Harwell stud male is shown through comparison with the C57BL/6J reference genome, with chromosome identifiers on the left. Each blue point represents a detected SNP at a given chromosome position. The *y*-axis of each chromosome plot shows the base-quality scores (BQS) for each SNP, ranging from 200 to 1000. The largest cluster of heterozygous SNPs was found on chromosome 13 between 38 and 76 Mb. Much smaller regions exhibiting a cluster of non-B6 SNPs could not be independently validated and were attributed to sequence context, sequencing errors or genetic drift. Note also the large number of SNPs on the Y chromosome, revealing differences between the *M. m. domesticus* Y^POS^ chromosome and the C57BL/6J Y chromosome.

The report of Arboleda *et al.* (2014), describing a dominantly acting modifier on chromosome 11 that confers protection against B6.Y*^POS^* sex reversal, prompted us to determine whether the Harwell B6.Y*^POS^* colony carried the same non-B6-derived region, possibly accounting for the presence of fertile males at each generation. It is the contention of Arboleda *et al.* that, in the absence of this region, the large majority of XY B6.Y*^POS^* mice would develop as females. Characterization of the appropriate SNPs indicated that the Harwell colony lacked this region (Table S1). We then performed whole-genome next-generation sequencing of a B6.Y*^POS^* stud male from the Harwell colony to determine whether any significant segments of its genome were not derived from B6. Bioinformatic analyses (see *Materials and Methods*) revealed that, in addition to the Y chromosome, an ∼37 Mb segment of chromosome 13 from ∼38.2 to 75.7 Mb contained a high density of non-B6 SNPs in the heterozygous state (Figure 1). Other, much smaller, non-B6 sequence clusters were detected, scattered throughout the genome, such as that detected on chromosome 4 (Figure 1). These could not be independently validated (data not shown); we interpret these as likely sequencing errors due to sequence context, such as structural or copy number variations indicated by abnormally high base coverage, or as variants that have arisen in the Harwell B6 colony and are not found in the B6 reference genome used to map the sequence reads.

**Figure 2:**
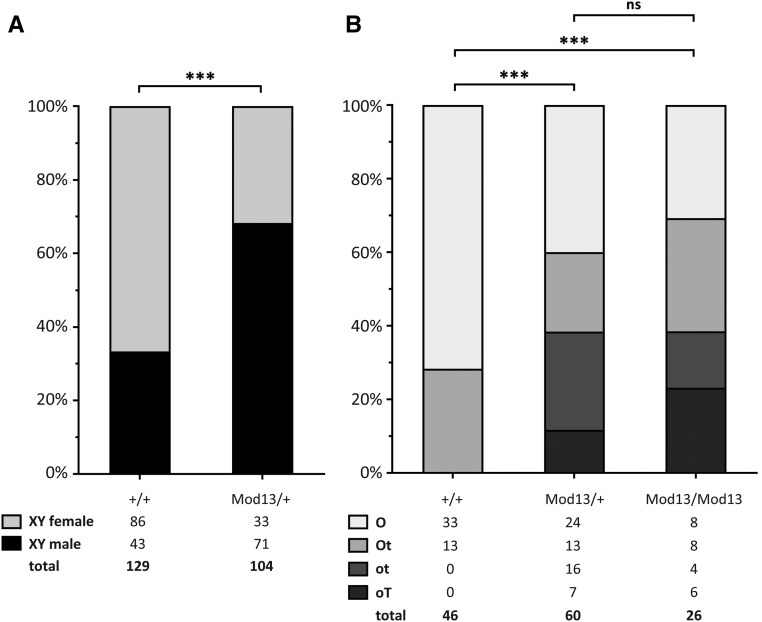
*Mod13* promotes male phenotypic sex and testis determination. (A) The proportions of mice with male or female phenotypic sex in cohorts of the Harwell B6.Y*^POS^* colony, grouped by genotype (+/+ and *Mod13*/+) following scoring at weaning; the numbers of mice in each group are shown beneath each bar; a significant difference was found in the male:female ratio between the two genotypic groups (***P < 0.0001, Fisher’s exact test). (B) Scoring of gonadal phenotype in B6.Y*^POS^* wild-type (+/+), heterozygous (*Mod13*/+), and homozygous (*Mod13*/*Mod13*) fetuses at 14.5 dpc. Gonads were scored as ovaries (O), OVOtestes (Ot), ovotestes (ot), or ovoTESTES (oT), depending on the proportion and quality of testicular tissue observed and the overall gonad shape and morphology; numbers of gonads in each group are indicated. B6.Y*^POS^*+/+ fetal gonads are significantly different to those in the *Mod13*/+ and *Mod13*/*Mod13* groups (***P < 0.0001, Fisher’s exact test). No normal testes (T) were observed in any B6.Y*^POS^* fetuses examined here.

We then sought to validate the existence of the non-B6 region on chromosome 13 by performing Sanger sequencing of PCR products encompassing nine putative SNPs identified across the region (Table S2), using all XY mice in the Harwell colony. This analysis confirmed the existence of these non-B6 SNPs, in the heterozygous form, in a proportion of mice in the colony (Figure 2). Moreover, while XY male mice were identified that lacked the region, and XY female mice were identified that carried the region, the non-B6 sequences were present at a significantly higher frequency in B6.Y*^POS^* male mice than in B6.Y*^POS^* female mice (Figure 2A). These data suggest that this region of chromosome 13 affords some degree of protection against B6.Y*^POS^* sex reversal. We called the region *Mod13*.

### *Mod13* influences primary sex determination in B6.Y*^POS^* mice

**Figure 3:**
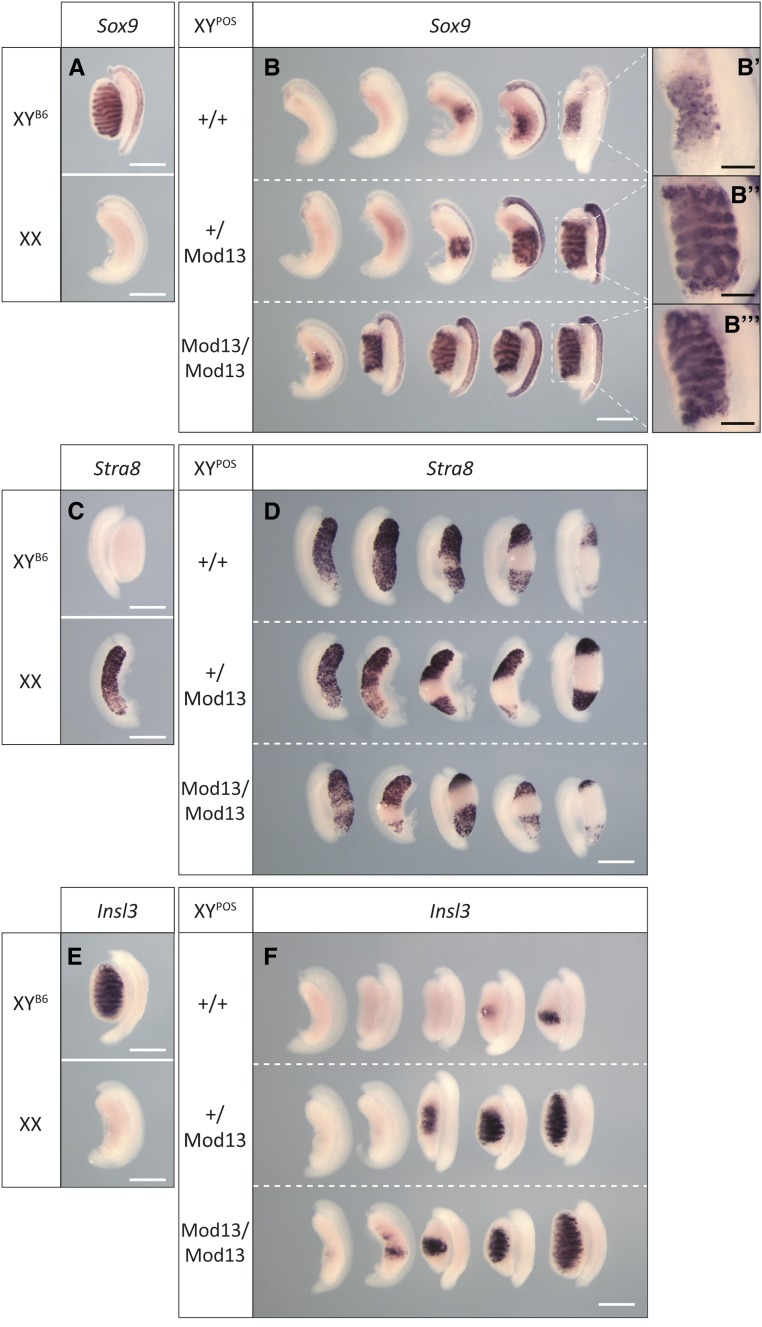
Cell marker analysis of the effect of *Mod13* on fetal XY gonad development using WMISH. (A, C, and E) Marker gene expression in control gonads, XY^B6^ and XX. (B) *Sox9* expression in B6.Y*^POS^* wild-type (+/+), heterozygous (*Mod13*/+) and homozygous (*Mod13*/*Mod13*) fetuses at 14.5 dpc. (B’–B’’’) Magnified images of relevant gonads showing details of testis cord morphology. (D) *Stra8* expression and (F) *Insl3* expression in the same genotypes as shown in B. White scale bar, 500 *μ*m; black scale bar, 200 *μ*m.

We then tested whether the presence of *Mod13* affects primary sex determination. We identified XY males and XX females carrying *Mod13* (*Mod13*/+), and performed timed-mates between these to generate B6.Y*^POS^* fetuses that were +/+, *Mod13*/+, and *Mod13*/*Mod13*. Remarkably, there was a clear effect of *Mod13* on the morphology of the XY fetal gonads at 14.5 dpc (Figure 2B and Figure 3). B6.Y*^POS^* fetuses lacking the region (+/+) formed ovaries or ovotestes with minimal *Sox9* expression (Figure 3, A and B), high levels of the meiotic germ cell marker *Stra8* (Figure 3, C and D), and little evidence of significant testis cord formation or Leydig cell differentiation, the latter judged on *Insl3* expression (Figure 3, E and F). *Mod13*/+ fetuses also developed ovaries, but there were larger numbers of ovotestes with significant testis cord formation (Figure 3, A–D). Finally, *Mod13*/*Mod13* homozygotes formed ovotestes with the greatest amount of testicular tissue (Figure 2B and Figure 3, A–D), including some gonads with clear evidence of high-quality cord formation throughout the majority of the tissue (Figure 3, B’–B’’’). These data indicate that the presence of *Mod13* influences the amount and quality of testicular tissue formed. We conclude from these data that *Mod13* affords protection against B6.Y*^POS^* sex reversal by enhancing the amount of testicular tissue formed in the fetal period. It behaves, therefore, like a classical quantitative trait locus: its presence is no guarantee of testis development and its absence is not a guarantee of complete gonadal sex reversal. Rather, it positively influences the probability of formation of testicular tissue and its morphological quality.

### *Mod13* positively affects *Sry* and *Sox9* expression and negatively affects *Rspo1* and *Foxl2* at the sex-determining stage of gonadogenesis

**Figure 4:**
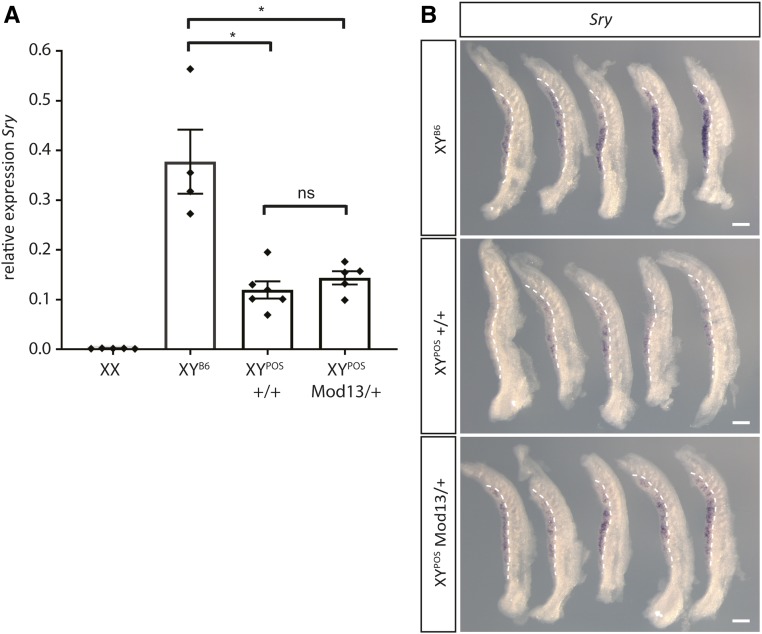
The influence of *Mod13* on *Sry* expression in B6.Y*^POS^* gonads. (A) qRT-PCR analysis of *Sry* expression in control (XX, *n* = 5, XY^B6^, *n* = 4) and B6.Y*^POS^* wild-type (+/+, *n* = 6) and heterozygous (*Mod13*/+, *n* = 5) fetuses at 16/17 tail-somite (ts) stage (11.25–11.5 dpc). Black diamonds denote individual gonad sample measurements. (B) WMISH analysis of *Sry* expression at the same stage and in the same XY genotypes as in A. *P < 0.05; two-tailed Welch’s *t*-test. ns, not significant. Bar, 200 *μ*m.

We first examined *Sry* and *Sox9* expression, two key genes in initiating testis development, in developing B6.Y*^POS^* gonads. *Sry* expression in B6.Y*^POS^* embryonic gonads has been reported to be delayed in comparison to B6.Y^B6^ gonads when analyzed by WMISH (Bullejos and Koopman 2005). We focused our analysis of *Sry* expression in B6.Y*^POS^*+/+ and *Mod13*/+ gonads on the 16 ts stage (∼11.25 dpc), at which point expression is reported to be close to its peak in B6.Y^B6^, but less advanced in B6.Y*^POS^* (Bullejos and Koopman 2005). qRT-PCR did not reveal any significant difference in *Sry* levels between the two genotypes (Figure 4A). However, *Sry* expression is especially spatiotemporally dynamic, and in our experience WMISH can offer a better indication of the progression of its expression. Because the variable phenotypes observed at 14.5 dpc, we analyzed five B6.Y*^POS^* gonads that were +/+ and five that were *Mod13*/+ by WMISH, to allow semiquantitative assessment of variation in expression levels and spatial patterns between distinct gonads. B6.Y^B6^ gonads were also included for comparison; these showed *Sry* expression throughout, although levels appeared to vary (Figure 4B). WMISH indicated that while a spectrum of *Sry* expression was observed within each B6.Y*^POS^* genotypic class, there was a clear tendency for expression to reach higher levels in *Mod13*/+ gonads, and for that expression to occupy a larger proportion of the gonad (Figure 4B).

**Figure 5:**
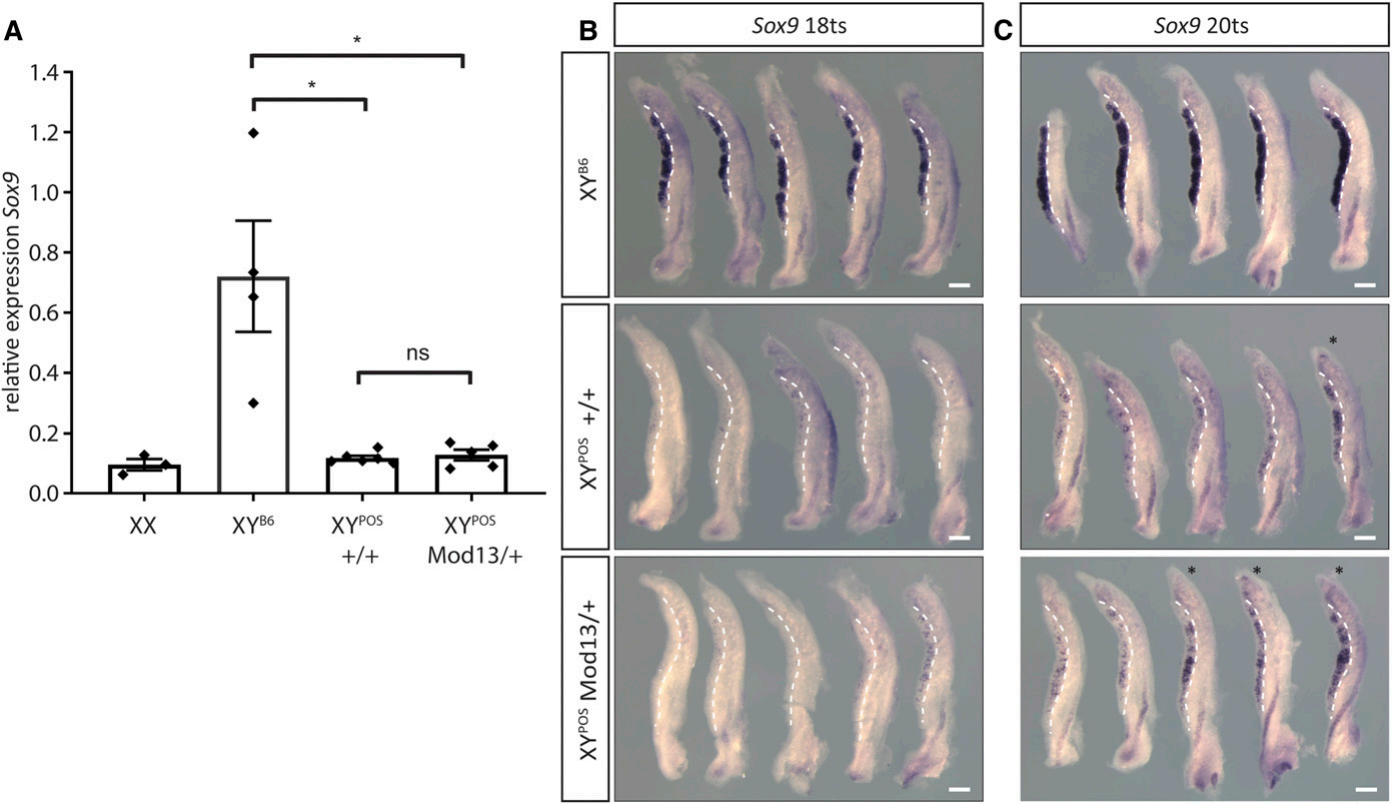
The effect of *Mod13* on *Sox9* expression at the sex-determining stage of gonad development. (A) qRT-PCR analysis of *Sox9* expression in gonads from control (XX, *n* = 3 and XY^B6^, *n* = 4) and B6.Y*^POS^* wild-type (+/+, *n* = 6) and heterozygous (*Mod13*/+, *n* = 5) fetuses at 16–17 tail-somite (ts) stage. Black diamonds denote individual gonad sample measurements. (B) WMISH analysis of *Sox9* expression in same XY genotypes as above, but at 18 ts stage. (C) WMISH analysis of *Sox9* expression in same XY genotypes as above, but at 20 ts stage. B6.Y*^POS^* gonads showing strong *Sox9* expression throughout most of gonad are marked by asterisks. Dotted lines indicate the gonad-mesonephros boundary, with gonad on the left. *P < 0.05; two-tailed Welch’s *t*-test. ns, not significant. Bar, 200 μm.

*Sox9* expression was then analyzed in B6.Y*^POS^*+/+ and *Mod13*/+ gonads using qRT-PCR and WMISH. At the 18 ts stage, expression was relatively weak in both genotypes, in contrast to strong expression in B6.Y^B6^ controls (Figure 5, A and B), possibly reflecting delayed *Sry^POS^* expression and the SRY^POS^ protein having reduced stability and/or functionality on B6 *Sox9* gonadal enhancers. However, at the 20 ts stage, WMISH indicated that *Sox9* expression was strong in three out of five *Mod13*/+ gonads; expression remained low in all but one of five +/+ samples (Figure 5C). *Sox9* expression was very strong throughout B6.Y^B6^ gonads at this stage (Figure 5C). These data are consistent with the positive effects of *Mod13* heterozygosity on testicular morphology observed at 14.5 dpc.

**Figure 6:**
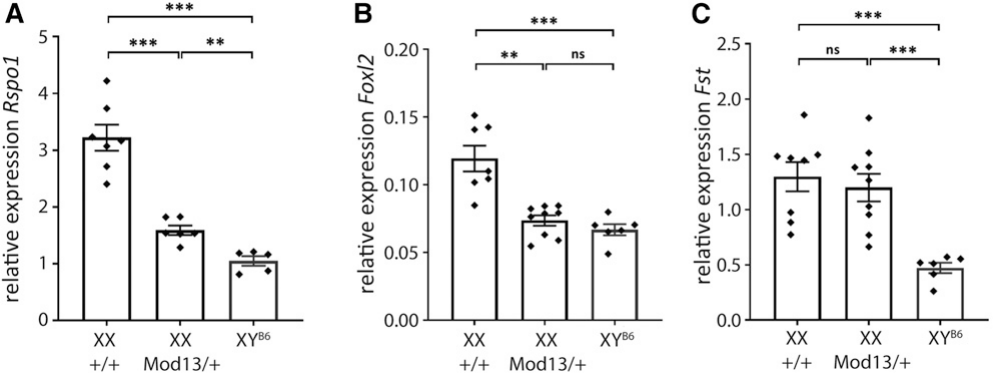
*Mod13* negatively affects pro-ovarian gene expression in XX gonads at 11.5 dpc. qRT-PCR analysis reveals expression levels of (A) *Rspo1*, (B) *Foxl2*, and (C) *Fst* in XX control (+/+), heterozygous B6.Y*^POS^* (*Mod13*/+) and control XY (XY^B6^) gonads at 11.5 dpc (17 tail-somite stage). **P < 0.005; ***P < 0.0005; two-tailed Welch’s *t*-test.

Given its positive effect on testis determination, we then examined whether the presence of *Mod13* affected ovary development in XX fetuses. First, analysis of XX+/+, *Mod13*/+, and *Mod13*/*Mod13* B6 gonads at 14.5 dpc with *Sox9* and *Stra8* using WMISH revealed no overt abnormalities at this stage (Figure S1A); nor was there any positive effect on *Sox9* expression in XX gonads at the sex-determining stage, 11.5 dpc, due to the presence of *Mod13* (Figure S1, B and C). To determine whether the presence of *Mod13* negatively affected the pro-ovarian pathway gene expression at the sex-determining stage, we carefully quantified expression of *Rspo1* and *Foxl2*, both markers of granulosa cell differentiation, in XX gonads that were either *Mod13*/+ or +/+. qRT-PCR revealed that levels of pro-ovarian *Rspo1* and *Foxl2* were significantly reduced in *Mod13*/+ gonads at 11.5 dpc, by ∼2-fold, to levels closer to those found in control XY gonads at the same stage (Figure 6, A and B); levels of follistatin (*Fst*) were not similarly affected by *Mod13* (Figure 6C). These reductions in *Rspo1* and *Foxl2* gene expression at 11.5 dpc, apparently not mediated by elevated *Sox9* in this context, are consistent with the overtly normal XX gonads observed at 14.5 dpc in *Mod13*/+ fetuses (see Figure S1). Mouse knockouts for both of these genes, and for *Wnt4*, only result in gonadal cell lineage reprogramming (sex reversal) in the perinatal/postnatal period (Pannetier *et al.* 2016). Nevertheless, the capacity of *Mod13* to reduce, perhaps transiently, expression of these genes at the critical sex-determining stage could, when allied to its positive effects on *Sry* and *Sox9* in the context of XY gonads, further enhance testis determination.

### Sequence comparisons indicate that *Mod13* has greater similarity to other inbred strains than C57BL/6J

**Figure 7:**
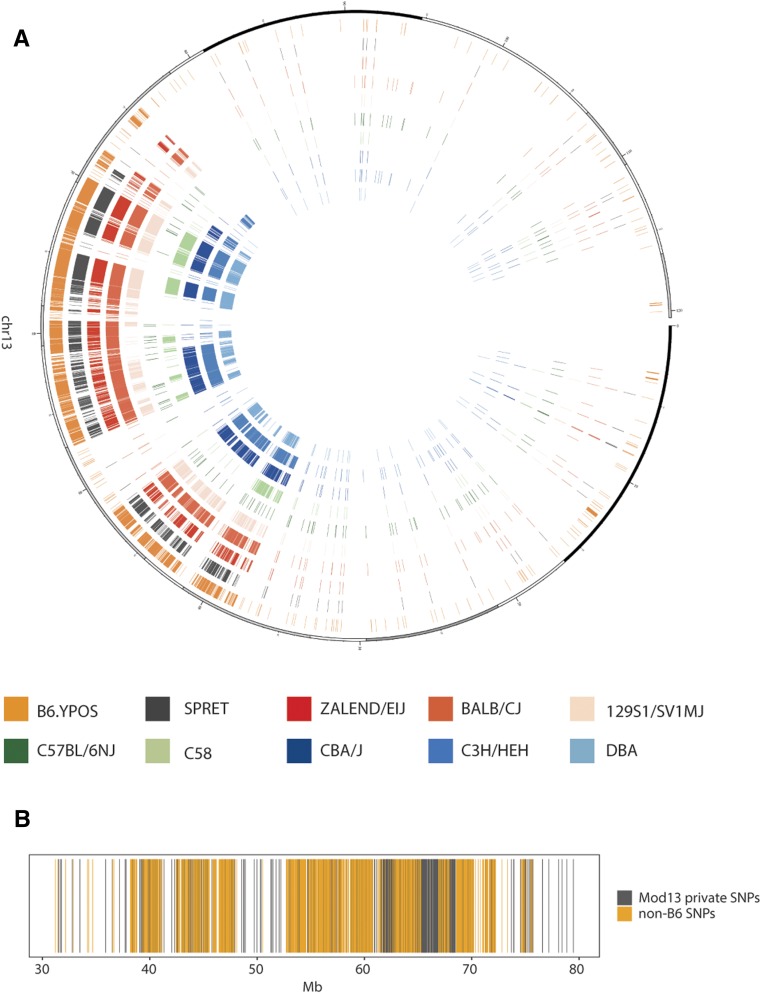
Heterozygous SNP density in *Mod13*. (A) Circos plot showing sequence comparisons between Harwell B6.Y^POS^ chromosome 13 and cognate sequences in nine other mouse strains. Chromosome 13 is represented as a circle, with Harwell B6.Y^POS^ sequences shown at the boundary and other genomes in concentric circles. Each line represents an individual SNP. Note the similarity between *Mod13* sequences (left) of Harwell B6.Y^POS^ and all of the other nine strains analyzed, with the exception of C57BL/6N. (B) Summary plot of SNP density grouped in 1 Mb windows over a 50 Mb interval containing the *Mod13* region of chromosome 13. Colors denote SNPs that are either private to *Mod13* when compared with 36 other mouse strains (gray) or are present in at least one other mouse strain (orange).

We performed a comparative analysis of chromosome 13 sequences, including the *Mod13* genomic region identified in the Harwell B6.Y^POS^ colony, in 36 laboratory mouse strains, comprising classical inbred strains and wild-derived mouse strains: a comparison between *Mod13* and nine of these is shown in Figure 7A. The B6.Y^POS^ *Mod13* region is complex, containing segments of B6 homozygosity in addition to heterozygous regions (Figure 7B), an organization that has presumably arisen by successive rounds of recombination over multiple generations of backcrossing to the B6 background. Remarkably, the non-B6 *Mod13* sequences showed significant similarity with most other inbred strains analyzed, with the greatest similarity to BALB/cJ; the exception was C57BL/6N (B6N), suggesting that B6J and B6N are both outliers in respect of sequence composition in this region of mouse chromosome 13 (Figure 7B). Moreover, the distribution of heterozygous SNPs across the region is not smooth: there are clusters of non-B6 SNPs as well as SNPs private to *Mod13*, where private is defined as SNPs found uniquely in *Mod13* (Figure 7B). These *Mod13* private SNPs cluster primarily between 60 and 70 Mb of chromosome 13 (Figure 7B).

### The non-B6-derived segments of *Mod13* contain no known primary sex-determining genes but contain genes expressed in supporting cells at 11.5 dpc

There are 268 protein-coding genes within the *Mod13* region, harboring 48 predicted mis-sense SNPs, from position 38.2 to 75.7 Mb of chromosome 13 (assembly GRCm38), which are not B6-derived (see Table S3). Only two of these genes have been previously implicated in sexual development: *Ptch1*, which is required for Leydig cell specification, but not testis determination (Clark *et al.* 2000; Yao *et al.* 2002); and *Adamts16*, which is linked to testis development in a number of contexts but is also not required for testis determination (Livermore *et al.* 2019). The known testis-determining gene *Gadd45g* (Warr *et al.* 2012) is located at position 51.8 Mb. However, the gene harbors no predicted deleterious SNPs and resides in a 4.5 Mb region of *Mod13* devoid of any significant clusters of non-B6 SNPs. We analyzed data from recent single-cell RNA-sequencing analyses of XY and XX gonads (Stévant *et al.* 2018, 2019) to determine whether any of these genes are expressed in the supporting cell lineage of the developing gonad (XY, XX, or both) at 11.5 dpc, a minimal requirement for a putative role in sex determination. No outstanding candidates were identified based on a combination of expression profile and biological link to the known sex-determining pathways, but a number of genes exhibit expression in supporting cell precursors at 11.5 dpc (Figure S2 and Table S3). Interestingly, the *Mod13* region does contain a number of key genes functioning in androgen biosynthesis, spermatogenesis, and male fertility (Table S3), such as *Srd5a1* (Mahendroo *et al.* 2001), *Hsd17b3* (Baker *et al.* 1997), and *Spata31* (Wu *et al.* 2015), which may promote fertility in B6.Y^POS^ *Mod13*/+ males independently of *Mod13*’s effect on primary sex determination.

## Discussion

Through the identification and phenotypic analysis of the variant *Mod13* region, we have established that the B6.Y*^POS^* sex reversal phenomenon is likely to have distinct genetic foundations depending on the breeding history of colonies held in different parts of the world. Interestingly, this possibility was predicted by Eicher and Washburn (2001), who suggested that an autosomal gene in a non-B6 state, which enhanced testicular development, would be kept in a forced heterozygous state by virtue of the breeding scheme used in B6.Y*^POS^* colonies (Eicher and Washburn 2001). This phenomenon is also consistent with the proposals of Arboleda *et al.* (2014), who predict that B6.Y*^POS^* colonies maintained by the selection of a transgene that protects against sex reversal, such as *Sry*, would, over time, lose modifiers that also enhanced the chances of male development, since they would be redundant in the presence of the transgene (Arboleda *et al.* 2014). However, they did not go on to predict the existence of distinct modifiers in geographically separated B6.Y^POS^ colonies. We have no definitive explanation for the origin of the Harwell *Mod13* variant of chromosome 13. The non-B6 sequence regions of *Mod13* do not precisely match any particular inbred strain, but sequence similarity to BALB/cJ is especially high. Moreover, all other inbred strains analyzed show similarity to *Mod13* sequences. Thus, sequence variants in this region are frequently unique to B6J and B6N, suggesting that these strains are outliers when it comes to sequence composition in this part of mouse chromosome 13.

It is our contention that one of following occurred: either *M. d. poschiavinus*-derived *Mod13* sequences were present in the earliest crosses performed when the Y*^POS^* chromosome was introgressed onto B6 and have been maintained by selection ever since, which cannot be tested given the absence of *M. d. poschiavinus* sequences in the mouse genome database; or, more likely, at some point after the establishment of the B6.Y*^POS^* strain at Harwell, an outcross was performed, to a strain thought likely to be protective against sex reversal, such as BALB/cJ, in an attempt to rescue a, perhaps small, colony in which very few B6.Y*^POS^* fertile males were being produced—this might be the fate of any colony lacking *Mod13* or another modifier/transgene. F1 males from such a cross would then have been crossed to B6 for subsequent generations, gradually resulting in the B6 background being restored, but with segments of the introgressed *Mod13* region being maintained. However, we can find no evidence of such an outcross in the breeding records at Harwell. In this context, it is interesting to note that several of the inbred strains showing sequence similarity to *Mod13* variants, such as BALB/cJ, C3H, and DBA, are known to protect against the disruptive effects of Y*^POS^*, *i.e.*, introgression of Y*^POS^* into these backgrounds does not overtly disrupt testis determination (Eicher *et al.* 1995). We hypothesize that the B6-unique region of chromosome 13 contributes to the particular sensitivity shown by this strain to testis determination defects.

We show that *Mod13* positively affects *Sry*, at least as evidenced by semiquantitative WMISH analysis, and *Sox9* expression at 11.25–11.5 dpc in XY gonads. *Sry* and *Sox9* levels appear enhanced, but variable, at this stage in XY *Mod13*/+ gonads, as predicted from the effects of *Mod13* on testis determination observed at 14.5 dpc. But the effect of *Mod13* is not restricted to this: we also report a negative effect on pro-ovarian expression at the same stage in XX gonads, not apparently mediated by changes to *Sox9* expression (nor, of course, *Sry*). Taken together, these data suggest a possibly complex, oligogenic effect of *Mod13* on testis determination during B6.Y*^POS^* gonadogenesis, simultaneously altering protestis and pro-ovary gene networks in direct and indirect fashion. We cannot, however, exclude the possibility that the positive effect on *Sry* expression is mediated by *Mod13*’s negative effect on pro-ovarian gene expression, by an unknown mechanism. Our data also indicate that the SRY*^POS^* protein can upregulate *Sox9* through the B6 version of the *Sox9* regulatory region, although some degree of functional mismatch might account for the apparent delay in *Sox9* reaching peak levels, even in the *Mod13*/+ B6.Y*^POS^* fetal gonad.

The large size of *Mod13* (∼37 Mb) suggests that there may be more than one causative gene responsible for its protective effect against XY gonadal sex reversal, with loci possibly distributed throughout the region, some with sequence variants directly promoting protestis pathways, and some opposing pro-ovarian pathways. Our data reveal an effect of *Mod13* on the amount and quality of testicular tissue formed in the fetal period, and it seems reasonable to conclude that this is a major determinant of the likelihood of adult males being fertile. *Gadd45g* would be an outstanding candidate for a gene exerting such effects, but no non-B6 variant clusters were detected in the region containing this testis-determining gene. We cannot rule out the possibility that the region also contains gene variants that promote male fertility, independently of effects on testis determination. In this context, it is interesting to note that the region contains genes such as *Srd5a1* and *Hsd17b3* that are implicated in adult male reproductive function.

Establishing causality for particular gene variants in the region is likely to be a daunting challenge. Our bioinformatic analysis indicates potential candidate genes on the basis of their expression in the supporting cell lineage at 11.5 dpc, the time at which, and cell lineage in which, sex determination is initiated in the mouse. None of these are previously implicated in sex determination, and each would need to be assessed for a role independently by inactivation and phenotypic assessment of the consequences. In addition to the possibility that multiple genes may play small, but additive, roles in testis determination in B6.Y^POS^ mice, it would be very difficult to identify particular *Mod13* sequence variants that are responsible for its protective effects. Some variants may not result in a loss-of-function effect, and so loss-of-function modelling may not reveal a role. *Mod13* is perhaps best viewed as a potential source of a novel sex-determining gene(s). In the meantime, the precise molecular basis of the B6.Y^POS^ sex reversal phenomenon remains unclear. We are now, however, able to examine B6.Y^POS^ sex reversal, and the effect of specific transgenes on this phenotype, in the absence of the confounding effects of *Mod13*.
